# Self-Cognizant Bionic Liquid Sensor for Pathogen Diagnosis

**DOI:** 10.34133/2021/9861513

**Published:** 2021-09-15

**Authors:** B. Fong

**Affiliations:** Providence University, Taiwan (Province of China)

## Abstract

As observed in the outbreaks of SARS and swine flu, as well as many other infectious diseases, the huge volume of human traffic across numerous enclosed public venues has posed immense challenges to preventing the spread of communicable diseases. There is an urgent need for effective disease surveillance management in public areas under pandemic outbreaks. The physicochemical properties associated with ionic liquids make them particularly suited for molecular communications in sensing networks where low throughput is quite adequate for pathogen detection. This paper presents a self-cognizant system for rapid diagnosis of infectious disease using a bionic sensor such that testing can be supported without collecting a fluid sample from a subject through any invasive methods. The system is implemented for testing the performance of the proposed bionic liquid sensing network.

## 1. Introduction

In recent years, the development of bionic sensing systems has led to the surge in application for infectious disease management [1]. Molecular solvents have been known to undertake a number of chemical reactions, making them particularly suited for molecular sensing applications through reactions of molecules in the solution phase [2]. Among a number of substances available for use as molecular sensing systems, ionic liquids utilizing ions' molten state with nonflammability, nonvolatility, and noncorrosiveness properties make molecular solvents particularly suited for ionic sensing [3]. Through the use of bionic sensing systems, specific sensing and communication networks can be designed upon such systems for a particular type of reaction since the solvent is composed of no less than two fragments; namely, the use of the organic cation part and either organic or inorganic anion part can represent different states, for example, to indicate either the presence or absence of a particular pathogen of interest. These solvents can therefore be designed to exhibit a specific set of properties through variation of a distinct end from either the cation or anion [4].

The use of triazole synthesis from a combination of azides and carbonyl compounds has been used in ionic sensing [5]. An earlier study by [6] has also studied sensing through three component reactions that yield 1,2,3-triazoles. Utilizing the properties of ionic liquids, this paper investigates the use of distinct ends from the cation and anion in the design and implementation of a self-cognizant sensing system [7], which can potentially solve the grand challenge of performing rapid diagnosis of viral infection in a mass scale.

This paper is organized as follows: Section 2 will provide an overview of the ionic sensing mechanism, followed by ionic liquids in rapid diagnosis in Section 3. Performance of the sensing system is evaluated using network performance analysis which is then presented in Section 4 before the paper is concluded in Section 5.

## 2. Ionic Liquid Sensing

**Figure 1:**
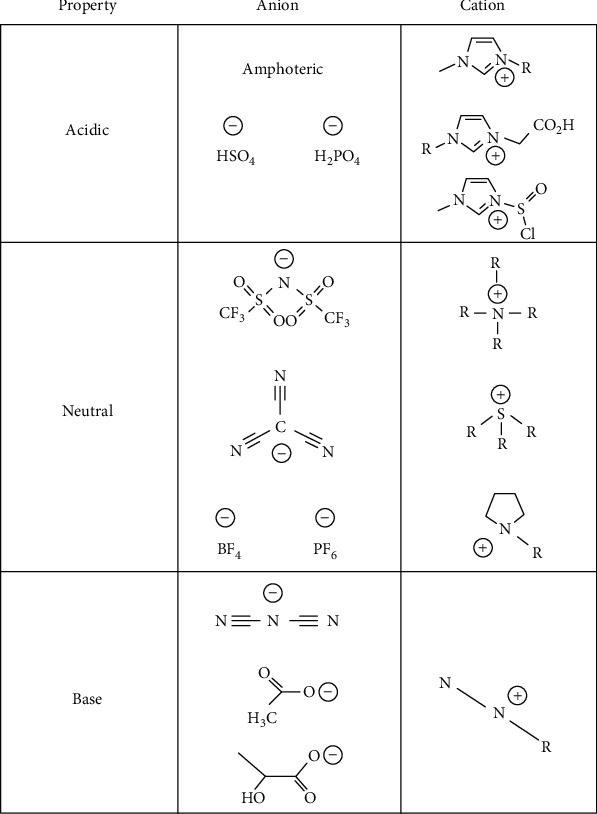
Ionic liquids' acid/base properties.

The preparation of ionic liquids commences with the Brønsted basicity principle across an air-water interface. This yields a thermally stable ionic liquid basic site with the cation as well as a basic anion [8]. Sensing can be accomplished by alteration of the base concentration given that ionic liquids exhibit the potential of modulating the basicity in base-mediated reactions [9]. An example of such is illustrated in Figure 1. Base-catalyzed condensation reactions are particularly suited for rapid diagnosis since the use of ionic liquids reduces the need for excess organic bases commonly used such as triethylamine and piperidine [10]. The catalyst can be selected to be reusable such as the use of [BMIM][OH] as both a catalyst and reaction medium [11].

An alternative ionic liquid is [DBU][Ac] (1,8-diazabicyclo[5.4.0]-undec-7-en-8-ium acetate), and aromatic/aliphatic amines are a catalyst that can undergo chemical reaction under solvent-free condition at room temperature [12]. One of the major use of any ionic liquids for sensing is to convey diagnosis results from a patient without physically carrying out invasive method of extracting fluid samples, which can be tested through synthesis of various heterocyclic and drug molecules that is relevant to the virus under test, for example, in the test of corona virus described in [13]. The procedure of bionic liquid synthesis of binary ionic liquid dialkylimidazolium chloroaluminate uses imidazolium moiety [14]. Changes in the alkyl substituent can yield the bionic liquid's desired physical properties. One of the important attributes of application in pathogen diagnosis is constituent ions' moisture sensitivity and basicity [15]. Acid base characteristics need to be considered because the reaction's effectiveness is, to a great extent, controlled by the reaction medium acidity and basicity [16] which is primarily determined by the ions' strength of the bionic liquid.

The process of synthesizing organic moieties that contain heterocycles is highly dependent on the specific moieties' chemical properties [17]. These organic moieties can be applied to serve in different sensing networks as they exhibit various potent biological properties against a number of diseases caused by cell mutation and neurological disorder as well as by different viruses [18].

The development of a noninvasive rapid diagnosis mechanism will entail new biological active molecules with appropriate therapeutic properties that are largely depending on the type of pathogen to be detected. More importantly, it must exhibit no toxicity to be used in any sensing system for diagnosis. Variants of heterocycles as well as 1,2,3-triazoles exhibit such potent biological activities for use as a base for ionic liquid sensing making them well suited to be used as therapeutic agents [19]. Moreover, 1,2,3-triazoles exhibit electronic properties of amide bonds as well as bioisosteric effects making it particularly suited to be used as the base for a pathogen sensing system [20].

### 2.1. 1,2,3-Triazole Base

**Figure 2:**
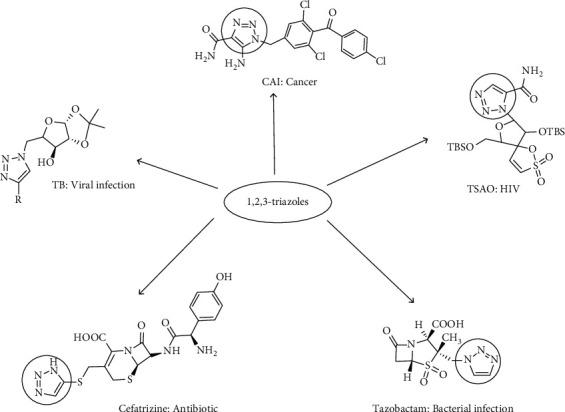
Biological properties of the triazole ring.

1,2,3-Triazoles exhibit pharmacophore biological properties against allergy being particularly appropriate for the detection of pathogens like bacteria and viruses [21] with such properties summarized in Figure 2. It serves as a therapeutic agent with properties such as strong dipole moment, higher stability, aromaticity, and amide bond for diagnosis through its dipole interactions with the pathogen [22].

**Figure 3:**
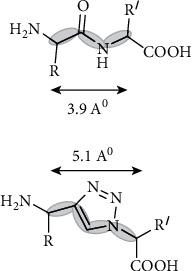
1,2,3-Triazole Z-trans-amide bond isostere.

1,2,3-Triazoles are antiretroviral protease synthetic molecule inhibitors used in combination for diagnosis as well as treatment as triazole with moieties possesses potent biological activities like antiviral, antiepileptic, antiallergic, antimicrobial, anticancer, and antituberculosis [23]. The particular form of the 1,2,3-triazole therapeutic agent that detects a specific biological target with dipole interactions and hydrogen bond is shown in Figure 3. Protease inhibitors bind to a viral enzyme thereby precluding pathogen from replicating making agents such as 1,2,3-triazoles particularly suitable for use as a therapeutic agent that exhibits antiviral activities and pharmacophore biological properties against allergy [24].

**Figure 4:**
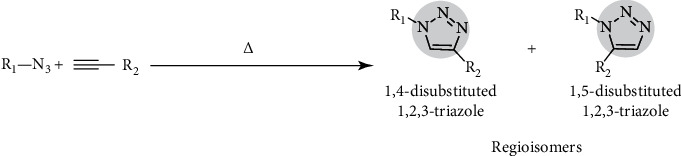
Thermally promoted cycloaddition yielding 1,4-disubstituted and 1,5-disubstituted 1,2,3-triazoles.

One of the main challenges of utilizing 1,2,3-triazoles as a base is that these molecules do not occur naturally such that they need to be synthesized [25]. The classical research by Bock et al. [26] documented thermally promoted cycloaddition of azide and alkyne reaction under varying conditions such that a copper salt selected as a prime catalyst Cu(I) can turn both azide and alkyne into 1,4-disubstituted 1,2,3-triazoles with at least a 90% product yield. This process is summarized in Figure 4.

### 2.2. Water-Based Diagnosis

Chemical reactions carried out in water are an important requirement for diagnosis in the context of safety of test subjects. [27] proposes a solid-phase synthetic reaction between azide and terminal alkyne that contains a peptide molecule by using a CuI/DIPEA catalyst such that CuAAC reactions are carried out in water, making it particularly suited in serving as a base for molecular communications within a human body for the purpose of carrying out diagnosis tests.

**Figure 5:**
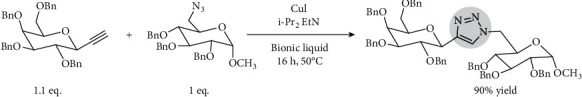
Sugar-linked 1,2,3-triazole synthesis.

To use water as solvent, carbohydrate azide and acetylenes are used to react under the catalyst of copper iodide (CuI), and different bionic liquids are screened for their reactions in order to carry out diagnosis tests as shown in Figure 5 such that the reaction can be carried out with an adequate yield.

## 3. A Safe Molecular Sensing Network

**Figure 6:**
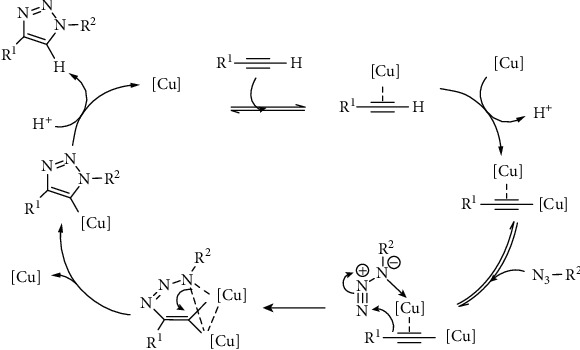
CuAAC reaction mechanism.

Basic sensing is supported by copper-catalyzed azide-alkyne cycloaddition reaction (CuAAC) through click reactions in a facile through a plausible mechanism that yields the copper acetylide and dinuclear complex as shown in Figure 6 that shows the mechanism of CuAAC reaction as the sensing mechanism.

**Figure 7:**
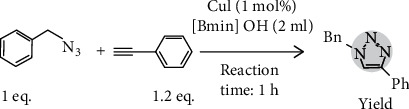
Reaction yields 1,4-disubstited 1,2,3-triazoles.

**Figure 8:**
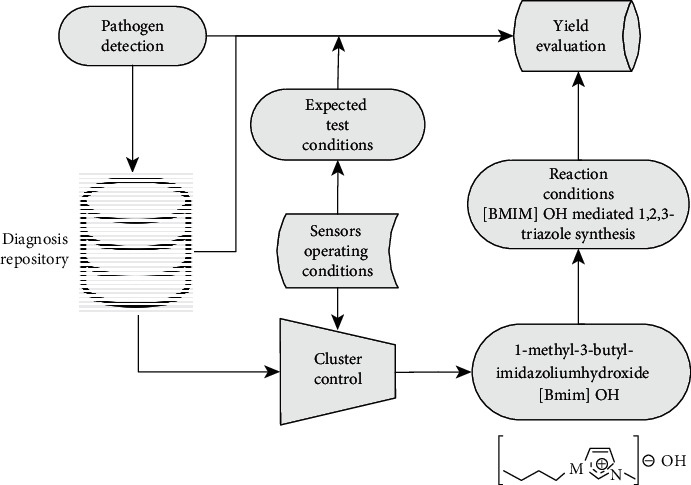
Test system architecture.

For the synthesis of 1,4-disubstituted triazoles from CuAAC reaction, the molecular sensing network relies on ionic liquid [Bmim]OH (1-methyl-3-butyl-imidazolium hydroxide) used in conjunction with CuI only, thereby eliminating the use of any base or reducing agent. Other additives are needed to serve two main purposes, namely, a reduction to take place within the reaction time duration and production of a cleaner liquid to enter the test subject's digestive system, since it is likely that a small amount of the substance will be swallowed. With both aromatic azide and aromatic terminal alkyne, it typically takes approximately 0.5 h reaction time for the product yield to reach 90% as shown in Figure 7; the [BMIM]OH-mediated 1,2,3-triazole synthesis illustrated here is subsequently used in our test mechanism as shown in Figure 8.

One of the major problems of relying on regioselective synthesis of 1,2,3-triazoles as a molecular sensing medium is due to copper's cytotoxic property being a limiting factor for its broader medical applications [28]. We therefore investigate the use of metal-free base-catalyzed [3 + 2] cycloaddition of azides with carbonyl compounds for synthesizing 1,2,3-triazole derivatives, without introducing any additional reaction time or the use of any hazardous organic solvents.

**Figure 9:**
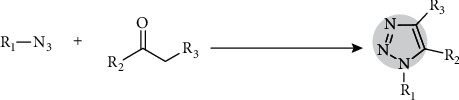
Cycloaddition of aryl azides.

Based on recent development by Gaetke and Chow [29], they reported 1,4,5-trisubstituted 1,2,3-triazole synthesis through cycloaddition of aryl azides with an active methylene compound. This provides an alternative copper-free substrate 4-nitrophenyl azide that reacts at room temperature with acetylacetone in ionic liquid [bmim]BF4. This would be apposite for rapid diagnosis as shown in Figure 9 with an active methylene compound. However, it does require a catalyst such as either Et_3_N or K_2_CO_3_. The reaction commences with initial formation of 1,3-dipolar cycloaddition of enolate ion under the presence of [bmim]OH. This reaction yields 1,4,5-trisubstituted 1,2,3-triazole through the elimination of water.

The regioselectivity is resulted by the azide's selective approach of positive terminal to the electron-rich part of the 1,3-dipolar cycloaddition's enolate ions [30]. We select Bu_4_NOH as a hydrated ionic liquid catalyst for its ease of availability and nonmetallic, nontoxic, and nonvolatile properties so that it can be used both efficiently and safely [31].

## 4. Performance Evaluation

Through utilizing the molecular sensing mechanism described above, rapid diagnosis can be supported by the use of a simple self-cognizant set of biosensor nodes that returns a very simple binary test result of either positive (presence of pathogen under test) or negative (absence of pathogen under test). Further, measurement of the location of each sensor node can provide insights into the concentration of pathogen, which can be particularly useful in the detection of asymptomatic carriers [32]. Each node therefore contains a node ID along with its location that can be transmitted through multihop communication to a sink node. A block diagram of the test system is shown in Figure 8. The purpose is to map the mutual distance between nodes [33]. We conducted a laboratory experiment to evaluate the use of bionic sensors for our performance evaluation on facile synthesis of 1,4-disubstituted triazoles through CuAAC reaction with bionic liquid [Bmim]OH (1-methyl-3-butyl-imidazolium hydroxide) in conjunction with CuI [34]. This has a reaction time that ranges between 0.5 and 1 h with both aromatic azide and aromatic terminal alkyne, yielding over 90%; this is in contrast to the use of aliphatic starting materials with a 3–10 h reaction time under similar yields [35].

Initially, a simple sensing network is set up with a cluster controller being selected in a distributed manner [36]. The network commences as being homogenous in terms of energy level. A cluster controller, one that effectively coordinates communications within a cluster of nodes, is selected according to the residual energy such that nodes that are active can serve as a cluster controller. Communication commences with the assumption that all nodes initially carry the same energy level *E*_*o*_.

The node that is selected as the cluster controller broadcasts a message across the entire network indicating that it is the controller of a specific cluster. Other nodes within the cluster will then make direct connection to this particular cluster controller. Clusters are formed on the basis of received signal strength of the received broadcast message from the cluster controller. An active node initiates a request to the nearest cluster controller when being grouped as within the same cluster. This effectively works as the Carrier Sense Multiple Access (CSMA) MAC protocol for collision avoidance [37]. The cluster controller conveys the residual energy information along with its distance from each node and selects an active node relaying information to the next hop [38]. An active node is selected from the maximum residual energy, and the cluster controller will establish a schedule for nodes within the cluster.

**Figure 10:**
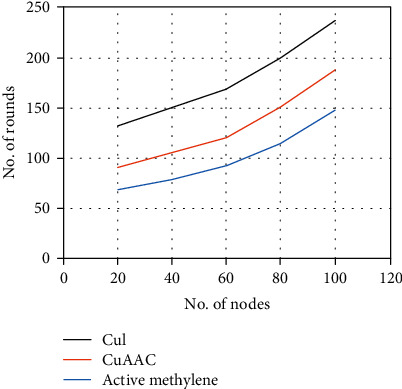
Network active time.

**Figure 11:**
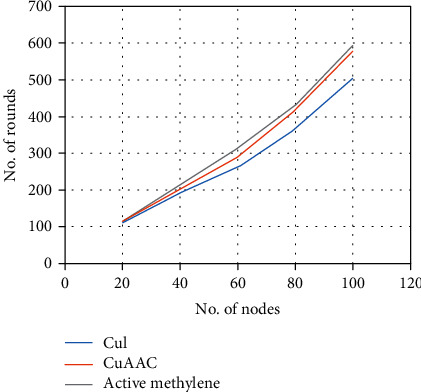
RTP performance.

To evaluate the bionic sensing system performance, we carry out simulation for up to 100 nodes with an equal initial energy level of *E*_*o*_ = 50 J. We base our evaluation on the Packet Transmission Ratio (PTR) that measures the ratio of redundant packets sent to the cluster controller from active node+to sink via the cluster controller so long as the network is active, i.e., as controlled by chemical reaction. The network active time performance is shown in Figure 10,

Cu(I) supports a higher maximum number of nodes acting as cluster controllers due to its longer response time. Consequently, the total energy, namely, the sum of *E*_*o*_, is the highest among the three possible implementations. CuAAC offers an improved overhead. The 1,4-disubstited 1,2,3-triazoles are obtained by reacting alkyl bromide, sodium azide, and terminal alkyne with a comparable 90% yield. The active methylene compound was used while the number of supported cluster controllers was reduced. The use of an appropriate clustering algorithm can save energy among individual nodes that consequently support the highest number of rounds.  Figure 11 also shows an enhancement in network active time with the active methylene compound. Redundant packets are also substantially reduced in the case of using the active methylene compound since there are fewer number of nodes communicating to the sink node compared to the other two implementations where both Cu(I) and CuAAC have more active nodes within the same cluster, making bionic sensing less efficient.

**Figure 12:**
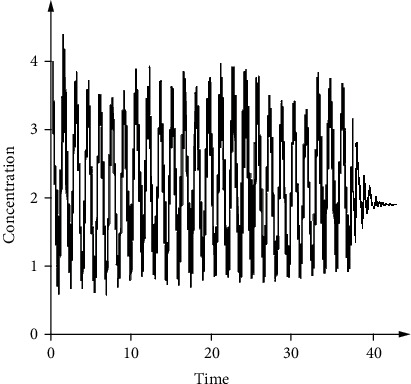
Output signal of the diagnostic system.

Finally, we test the prototype using the BioSD architecture proposed by Hussain et al. [39] as the chemical reaction network (CRN). The main objective is for the realization of signal differentiation for rapid diagnosis [40]. The output is proportional to the input's derivative in the signal differentiator such that the biomolecular specimen concentration will be a representation of the presence of pathogen in the diagnosis test. The sample test output signal is shown in Figure 12. When correctly implemented, this output signal should correspond to the rate of change of the input signal. This output shows that the steady state of each derivative action is achieved at approximately 43 seconds. In actual diagnostic test implementation, noise reduction can help increase the performance of the BioSD network [41].

## 5. Conclusion

This paper discusses the design of a molecular bionic sensor based on the synthesis of 1,4,5-trisubstituted-1,2,3-triazoles using various ionic liquids as catalyst as well as reaction medium without the need of an external base. The use of azide-alkyne cycloaddition and cycloaddition of azides with active methylene compounds is particularly suited to serve as a bionic sensing system for rapid diagnosis in the presence of selected pathogens, owing to its nonmetallic and nontoxic base properties with short reaction time and adequate yields.
